# Estimating the Health Care Expenditure to Manage and Care for Type 2 Diabetes in Nepal: A Patient Perspective

**DOI:** 10.1177/23814683231216938

**Published:** 2023-12-14

**Authors:** Padam Kanta Dahal, Lal Rawal, Zanfina Ademi, Rashidul Alam Mahumud, Grish Paudel, Corneel Vandelanotte

**Affiliations:** School of Health, Medical and Applied Sciences, Central Queensland University, Sydney Campus, Sydney, NSW, Australia; Appleton Institute, Physical Activity Research Group, Central Queensland University, Queensland, Australia; School of Health, Medical and Applied Sciences, Central Queensland University, Sydney Campus, Sydney, NSW, Australia; Appleton Institute, Physical Activity Research Group, Central Queensland University, Queensland, Australia; Translational Health Research Institute (THRI), Western Sydney University, Sydney, NSW, Australia; Centre for Medicine Use and Safety, Faculty of Pharmacy and Pharmaceutical Sciences, Monash University, Melbourne, Australia; School of Public Health and Preventive Medicine, Monash University, Melbourne, Australia; NHRMC Clinical Trials Centre, Faculty of Medicine and Health, The University of Sydney, Camperdown, NSW, Australia; School of Health, Medical and Applied Sciences, Central Queensland University, Sydney Campus, Sydney, NSW, Australia; Appleton Institute, Physical Activity Research Group, Central Queensland University, Queensland, Australia; Appleton Institute, Physical Activity Research Group, Central Queensland University, Queensland, Australia

**Keywords:** cost estimation, direct and indirect medical cost, health care cost, out-of-pocket payment, type 2 diabetes

## Abstract

**Background.** This study aimed to estimate the health care expenditure for managing type 2 diabetes (T2D) in the community setting of Nepal. **Methods.** This is a baseline cross-sectional study of a heath behavior intervention that was conducted between September 2021 and February 2022 among patients with T2D (*N* = 481) in the Kavrepalanchok and Nuwakot districts of Nepal. Bottom-up and micro-costing approaches were used to estimate the health care costs and were stratified according to residential status and the presence of comorbid conditions. A generalized linear model with a log-link and gamma distribution was applied for modeling the continuous right-skewed costs, and 95% confidence intervals were obtained from 10,000 bootstrapping resampling techniques. **Results.** Over 6 months the mean health care resource cost to manage T2D was US $22.87 per patient: 61% included the direct medical cost (US $14.01), 15% included the direct nonmedical cost (US $3.43), and 24% was associated with productivity losses (US $5.44). The mean health care resource cost per patient living in an urban community (US $24.65) was about US $4.95 higher than patients living in the rural community (US $19.69). The health care costs per patient with comorbid conditions was US $22.93 and was US $22.81 for those without comorbidities. Patients living in rural areas had 16% lower health care expenses compared with their urban counterparts. **Conclusion.** T2D imposes a substantial financial burden on both the health care system and individuals. There is a need to establish high-value care treatment strategies for the management of T2D to reduce the high health care expenses.

**Highlights:**

- More than 60% of health care expenses comprise the direct medical cost, 15% direct nonmedical cost, and 24% patient productivity losses. The costs of diagnosis, hospitalization, and recommended foods were the main drivers of health care costs for managing type 2 diabetes.
- Health care expenses among patients living in urban communities and patients with comorbid conditions was higher compared with those in rural communities and those with without comorbidities.
- The results of this study are expected to help integrate diabetes care within the existing primary health care systems, thereby reducing health care expenses and improving the quality of diabetes care in Nepal.

## Graphical abstract

**Graphical Abstract:**
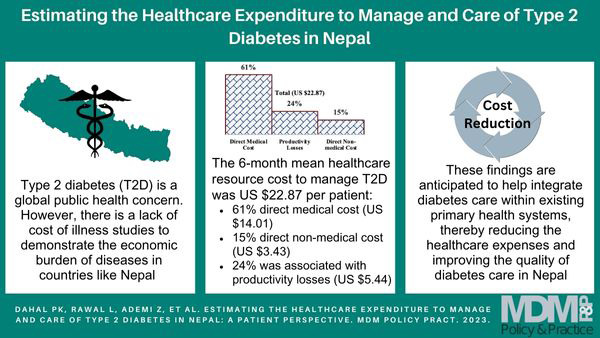
This is a visual representation of the abstract.

Globally, type 2 diabetes (T2D) is identified as a major public health concern, placing a heavy burden on individuals’ health and socioeconomic development.^1^ It is estimated that more than 537 million people (age 20–79 y) are living with diagnosed T2D, and a large portion remained undiagnosed.^2,3^ The International Diabetes Federation (IDF) estimated that 783 million people will be living with diabetes by 2045.^2^ Moreover, it is projected that most (79%) people with T2D will live in lower- and upper-middle-income countries and that most will remain undiagnosed.^2^ T2D is one of the most burdensome and costly health conditions, imposing a substantial financial burden to society.^2,3^ In 2021, US $966 billion was spent on diabetes, and by 2030, this expenditure is expected to reach pandemic levels, with costs growing to more than US $2.1 trillion.^2,4^

Nepal is also facing the challenges of chronic diseases including T2D. A recent nationwide population-based survey and IDF reported that the prevalence of diabetes mellitus was 8.5% and 8.7% respectively, and it is expected to reach 9.4% by 2045.^5,6^ The total health care expenditure, including out-of-pocket expenses, to manage T2D in Nepal was US $115.8 million and US $102.2 per person per year in 2021 and is projected to nearly double (i.e., total cost US $190.5 million), with costs of US $168.1 per person by 2045.^6^ Over half of the expenses of Nepalese T2D patients is out-of-pocket and is becoming a major concern in low and middle income countries like Nepal.^7,8^

Despite T2D being a major concern for Nepal, there is a lack of cost-of-illness studies to demonstrate the economic burden of diseases in the health care system and society. To the best of our knowledge, no other study has estimated the health care resource use costs of T2D in Nepal. Therefore, this study aimed to estimate the health care resource use expenses for patients managing T2D and to identify whether and to what extent socioeconomic, demographic, and behavioral factors had an impact on health care expenditure in Nepalese community settings. Information derived from this study can support decision makers in setting priority for health care policies and health care programs.

## Methods

### Study Design, Setting, and Participants

This study is a baseline descriptive cross-sectional study of a health behavior intervention conducted in the Kavrepalanchok and Nuwakot districts of Nepal. It is a community-based study conducted among people with T2D (aged 30–70 years) living in the Kavrepalanchok and Nuwakot districts of Nepal. The data were collected between September 2021 and February 2022.

### Sample Size

For this study, a sample of 481 patients with T2D were interviewed from the selected districts of Nepal using a structured questionnaire. The sample size was estimated considering the prevalence of the outcome variable for this study (i.e., the proportion of health care expenses to manage T2D).^9^ Briefly, the estimated sample needed was 385 at 5% marginal error. However, considering the additional 20% of patients needed to allow adjustment of losses to interviews, withdrawals, and incomplete information during the data collection process, the ultimate sample size needed was 481.

### Inclusion and Exclusion Criteria

Adults aged 30 to 70 y who were clinically diagnosed with T2D were included in the study. People with clinically diagnosed type 1 diabetes mellitus, currently pregnant, and critically ill patients who had no ability to participate in the program were excluded from this study.

### Cost Measurement

This study estimated the cost from the health care system and adheres to the Consolidated Health Economic Evaluation Reporting Standards 2022 (CHEERS) statement^10^ (see Supplementary File, Table 1). The development and validation of the tools to measure the health care expenses were based on the guidelines proposed by Laberge et al.^11^ To generate the unit cost of resource use, the study by Curtis and Burns was used.^12^ Moreover, this study also adhered to the Strengthening The Reporting of Observational Studies in Epidemiology (STROBE) statement checklist^13^ (see Supplementary File, Table 2). The average cost per person was estimated using the following resources: diagnostic, medication, health specialist visits, hospitalization, and transaction costs (i.e., accommodation, transportation, and food items). All costs were calculated in Nepali rupees (NRs) and converted to US dollars based on the average exchange rate of 2021 and adjusted using the 2021 consumer price index for Nepal (i.e., US $1 = NRs 118.13).^14^

### Health Care Resource Use

To estimate health care resource use, we considered direct medical costs and direct nonmedical costs. Direct medical costs include diagnostic and health service utilization costs (clinical visits, medications, and hospitalizations). These costs were based on the services price list implemented by Dhulikhel Hospital. This is because Dhulikhel Hospital is located in the semi-urban community of the Kavrepalanchok district, which operates multiple outreach health centers in the community settings of the Kavrepalanchok and Nuwakot districts. Furthermore, most patients in our study rely heavily on the services provided by this hospital, which serves as a primary service provider and referral center for these communities. Direct nonmedical costs include transportation, food, and accommodation, and indirect costs include time spent traveling to service or loss of work while seeking health care services. Health care resource utilization was identified based on the patient’s expenditure of hospital visits, prescribed medications, hospital stay, and medical screening tests within the past 6 months. Outpatient and inpatient health expenditures within the past 6 mo in both private and public health care centers and health care expenses were assessed (see Supplementary Table 3).

#### Diagnostic costs

Diagnostic costs were identified from the cost of each test to screen for T2D, which was obtained from the service price list implemented by Dhulikhel Hospital, Nepal. The listed price by this hospital was considered as a standard price list to estimate the screening cost at the selected districts of this study.

#### Medication costs

Uses of medicine (i.e., metformin, Glycomet, Diapride, Effimet, formin, gliclazide, Zoryl-1, etc.) and their dosages for managing T2D were evaluated, as well as the use of medication for comorbidities such as heart disease, difficulty in breathing, anxiety/depression, blood cholesterol control, blood clot prevention, and high blood pressure. The price of the medicine was obtained from Dhulikhel Hospital, Nepal, and converted based on the medication used by the patient.

#### Health specialist visits

Health specialist costs for cardiologists, ophthalmologists, dentists, medical officers, and health assistants in both public and private facilities were considered to calculate the health specialists’ rate per visit. Dhulikhel Hospital’s outpatient department charges and specialist fees were considered to calculate the total health specialists’ costs.

#### Hospitalization cost

The charge of separate number admissions for hospital stays, overnight stay, and total length of stay were considered to estimate the hospitalization cost using the Dhulikhel Hospital charges as a reference.

#### Food items cost

Only consumption of fruits and vegetables were considered as an estimate for the food items cost, because a higher intake of fruits and vegetables is an important part of the diet associated with a lower risk of having T2D.^15^ The calculation of food item costs was based on the number of servings of fruits and vegetables consumed per day and the local market price at the time of data collection.^16^ One serving of fruit contains 1 medium-sized fruit such as an apple or 2 small-sized fruits, and a half cup of cooked vegetables or 1 cup of salad was considered as a serving of vegetables.^17–19^

#### Transportation cost

Transportation time and cost were identified from the distance needed to travel to the nearest health facility. The listed prices for local travel were obtained from the Government of Nepal, Ministry of Physical Infrastructure and Transport, Department of Transport Management.^20^

#### Cost per hour lost

The human capital approach was applied to calculate hours lost (i.e., patient income losses). This approach is used to estimate the value of human capital as the current value of his or her earnings as a proxy for future productivity.^21^ The time was valued based on the minimum unskilled labor wage provided by the Nepal Government, which is US $127.98 (NRs 15,000) per month and US $ 0.65 (NRs 77) per hour.^14,22^ This is because the study participants were based in a community where the main source of income is agriculture. Indirect costs (i.e., income losses) for each inpatient’s hospital stay were estimated to be 8 h of paid work, and each outpatient visit was assumed to be 4 h of paid work. The transportation time was calculated and converted into hour loss expenses.

This study considered the bottom-up and micro-costing approaches for the cost estimation that allowed for a more precise assessment of the health care cost, which helped to identify the cost to the patients directly and provided insight into the patient’s subgroups, which was helpful for assessing local cost variation.^23,24^

### Statistical Analyses

Collected data were checked for accuracy (i.e., cleaning, coding, and processing) by the research assistants under the supervision of investigators and research officers. Characteristics of the participants were reported using frequencies and percentages for categorical variables, whereas means and standard deviations were used for continuous variables. Resource use costs were summarized by mean and standard deviation. An independent-sample *t* test was used to compare the mean health care resource use and its cost between residency status (i.e., urban and rural) and comorbidity. The 95% confidence intervals for these comparisons were obtained.

A generalized linear model, gamma with a log-link function, was used for modeling the continuous right-skewed total costs per patient. Independent categorical variables (i.e., gender, ethnicity, marital status, education, employment, religion, residency, comorbidity, year of living with T2D, insurance status, and monthly family income quintile) were considered as factors, and independent continuous variables (i.e., age) were considered covariants. The model was obtained by the main effect estimation method for selection of independent variables. We calculated the beta coefficient for every level of categorical variable, a single beta coefficient for a continuous variable, probability values (i.e., *P* value), and 95% confidence intervals (CIs). Variables with a *P* value of ≤0.20 in the unadjusted model such as ethnicity, marital status, education, employment, residency status, and monthly family income quintile were adjusted in the analyses to avoid missing important variables and to provide a more accurate picture for the underlining associations. Furthermore, the variance inflation factor (VIF) test was performed to determine the multicollinearity of independent variables. In line with accepted practice, a threshold VIF value of <5 was adopted, suggesting no evidence of multicollinearity between independent variables encompassed within the model. In addition, sensitivity analyses were performed to obtain 95% CIs by using the bootstrapping resampling (*n* = 10,000) technique, which helps to estimate the uncertainty and variability associated with the small-sample, nonlinear, and nonnormal data and complex statistical models.^25^ All statistical analyses were 2 sided, and *P* < 0.05 was considered to be statistically significant. Data were cleaned and coded in Microsoft Excel, and statistical analysis was performed using SPSS version 28.

## Results

### Baseline Characteristics of the Participants

The mean age of the participants was 54.4 ± 9.4 years, and 52.8% were male. Fewer than half of participants (45.3%) indicated that they had basic education (primary and secondary levels), followed by 36.5% who indicated that they did not attain any education. Agriculture (42.8%) was the main occupation, followed by being a housewife (22.0%), having a personal business (15.2%), and delivering services (8.1%). Most (90%) participants were Hindu, almost two-thirds (64.2%) were living in urban areas, and 49.5% indicated that they had chronic comorbid or multimorbid conditions (i.e., coronary heart disease, dyspnea, depression/anxiety, hypertension, thrombosis, hypercholesteremia; Table 1).

**Table 1:** General Characteristics of the Participants

Parameter	Frequency (*N* = 481)	Percentage
Mean age (*s*), y	54.44 (9.42)	
Gender		
Male	254	52.81
Female	227	47.19
Ethnicity		
Brahmin	187	38.90
Newar	115	23.91
Janajati (Magar/Tamang/Rai/Limbu)	93	19.33
Chettri (Thakuri/Sanyasi)	53	11.00
Others (Kami/Damai/Sarki/Gaaine/Baadi/Shah/Tharu/Jha)	33	6.86
Marital status		
Married	447	92.93
Others (never married, separated, and widowed)	34	7.07
Education status		
No education	176	36.59
Basic (primary and lower secondary)	218	45.32
Intermediate (upper secondary and postsecondary nontertiary)	36	7.48
Tertiary (university level)	28	5.82
Informal	23	4.78
Employment status		
Agriculture/animal husbandry	206	42.83
Housewife	106	22.04
Business	73	15.18
Services	39	8.10
Others (driver/labor (trained/untrained)/retired/household chores)	57	11.85
Religion		
Hindu	429	89.19
Buddhist	38	7.90
Christian	14	2.91
Residency		
Urban	309	64.24
Rural	172	35.76
Comorbidity		
Yes	238	49.48
No	243	50.52
Years of living with T2DM		
Less 1 year	124	25.78
More than 1 year	357	74.22
Insurance status		
Yes	214	44.50
No	267	55.50
Monthly family income quintile (US $), *n* (%)		
Poorest (≤84.65)	82	17.05
Second quintile (84.66–126.98)	114	23.70
Third quintile (126.99–228.56)	91	18.92
Fourth quintile (228.57–423.26)	86	17.88
Richest quintile (423.27 and above)	108	22.45

NRs, Nepali rupees; *s*, standard deviation; T2DM, type 2 diabetes mellitus; US $, United States dollar.

### Volume of Resource Use and Costs

Mean medicine use was 1.57 ± 1.87 times per 6 mo per patient followed by diagnostic (1.53 ± 1.73) and medical consultations (0.098 ± 0.65). The mean length of stay in hospital was 8.60 ± 6.04 d, followed by waiting time (1.07 ± 1.05 h) and travel time to the nearest health facility (0.93 ± 1.24 h; Table 2).

**Table 2:** Volume of Resource Use per Patient to Manage Type 2 Diabetes Mellitus^a^

Resource	Item	Overall Mean (*s*); (*n* = 481)	Rural Mean (*s*); (*n* = 172)	Urban Mean (*s*); (*n* = 309)	Mean Difference (95% CI)	*P* Value	Comorbid (*s*); (*n* = 238)	No Comorbid (*s*); (*n* = 243)	Mean Difference (95% CIs)	*P* Value
Medical consultation	Medical officer/general practitioner (times)	0.06 (0.23)	0.04 (0.20)	0.06 (0.24)	−0.02 (−0.06; 0.02)	0.27	0.06 (0.24)	0.05 (0.23)	0.01 (−0.04; 0.05)	0.80
	Cardiologist (times)	0.01 (0.10)	NA	0.013 (0.11)	NA	NA	0.01 (0.09)	0.01 (0.09)	0.00 (−0.02; 0.02)	0.98
	Ophthalmologist (times)	0.02 (0.16)	0.02 (0.13)	0.03 (0.17)	−0.01 (−0.04; 0.02)	0.46	0.04 (0.19)	0.01 (0.11)	0.03 (−0.003; 0.05)	0.075
	Dentist (times)	0.002 (0.05)	NA	0.003 (0.06)	NA	NA	0.004 (0.06)	NA	NA	NA
	Podiatrist (times)	0.002 (0.05)	NA	0.003 (0.06)	NA	NA	NA	0.00 (0.06)	NA	NA
	Health assistant (times)	0.004 (0.06)	NA	0.01 (0.08)	NA	NA	0.004 (0.06)	0.004 (0.06)	0.00 (−0.01; 0.01)	0.99
Diagnostic	HbA1c (times)	0.58 (0.49)	0.47 (0.50)	0.65 (0.48)	−0.19 (−0.28; −0.09)	<0.001	0.60 (0.49)	0.57 (0.49)	0.03 (−0.06; 0.12)	0.47
	Electrocardiogram (times)	0.17 (0.38)	0.13 (0.34)	0.19 (0.40)	−0.07 (−0.13; 0.001)	0.052	0.19 (0.39)	0.15 (0.36)	0.04 (−0.03; 0.10)	0.28
	Dilated eye examination (times)	0.62 (0.49)	0.52 (0.50)	0.67 (0.47)	−0.15 (−0.24; −0.06)	<0.001	0.66 (0.48)	0.58 (0.49)	0.08 (−0.01; 0.17)	0.073
	Foot care examination (times)	0. 16 (0.37)	0.12 (0.32)	0.18 (0.39)	−0.07 (−0.13; 0.000)	0.049	0.16 (0.36)	0.16 (0.37)	−0.005 (-0.07; 0.06)	0.88
Medicine	Diabetes mellitus (times)	0.94 (0.24)	0.94 (0.24)	0.94 (0.24)	0.00 (−0.04; 0.05)	0.88	0.94 (0.24)	0.94 (0.23)	−0.01 (−0.05; 0.04)	0.80
	Coronary heart disease (times)	0.04 (0.19)	0.03 (0.17)	0.05 (0.21)	−0.02 (−0.05; 0.02)	0.38	0.08 (0.27)	NA	NA	NA
	Dyspnea (times)	0.02 (0.15)	0.02 (0.15)	0.02 (0.15)	0.00 (−0.03; 0.03)	0.97	NA	NA	NA	NA
	Depression/anxiety (times)	0.01 (0.12)	0.01 (0.11)	0.02 (0.13)	−0.01 (−0.03; 0.02)	0.69	0.03 (0.17)	NA	NA	NA
	Hypercholesterolemia (times)	0.14 (0.35)	0.16 (0.37)	0.13 (0.34)	0.03 (−0.04; 0.10)	0.37	0.29 (0.46)	NA	NA	NA
	Hypertension (times)	0.36 (0.48)	0.32 (0.47)	0.39 (0.49)	−0.07 (−0.16; 0.02)	0.130	0.74 (0.44)	NA	NA	NA
	Thrombosis (times)	0.01 (0.11)	0.01 (0.08)	0.02 (0.13)	−0.01 (−0.03; 0.008)	0.26	0.03 (0.16)	NA	NA	NA
	Others (high fever, common cold, pain relief) (times)	0.05 (0.23)	0.06 (0.24)	0.05 (0.22)	0.01 (−0.04; 0.05)	0.77	0.05 (0.23)	0.05 (0.23)	0.00 (−0.04; 0.04)	0.96
Hospitalization	Separate number of admissions (times)	1.40 (0.69)	1.67 (0.58)	1.29 (0.76)	0.38 (−0.76; 1.52)	0.46	1.00 (0.00)	1.67 (0.81)	−0.67 (−1.52; 0.19)	0.102
	Overnight stay (days)	0.02 (0.14)	0.02 (0.13)	0.02 (0.15)	−0.01 (−0.03; 0.02)	0.70	0.02 (0.13)	0.02 (0.16)	−0.01 (−0.03; 0.02)	0.55
	Length of stay (LOS) days	8.60 (6.04)	8.00 (6.56)	8.86 (6.34)	−0.86 (−11.03; 9.31)	0.85	9.25 (5.74)	8.17 (6.74)	1.08 (−8.41; 10.58)	0.80
Transportation	Travel time (hour)	0.93 (1.24)	1.14 (1.56)	0.67 (0.92)	0.75 (0.49; 1.003)	<0.001	0.95 (1.26)	0.92 (1.22)	0.03 (−0.19; 0.25)	0.82
Food items	Recommended fruits & vegetables (times)	3.63 (2.69)	3.64 (2.67)	3.63 (2.70)	0.02 (−0.51; 0.54)	0.95	3.76 (2.64)	3.50 (2.74)	0.26 (−0.25; 0.77)	0.32

NA, not reported or not applicable; *s*, standard deviation.

a *P* value was obtained from an independent-sample *t* test.

### Health Care Costs

The total direct expenses of health care resource use to manage T2D was US $22.87 ± 16.68 per patient. Within the total cost, the mean diagnostic cost per patient was higher (i.e., US $8.55 ± 6.40) compared with hospitalization (US $4.84 ± 6.37), use of recommended food items (US $2.82 ± 2.40), transportation (US $0.61 ± 0.81), medical consultation (US $0.51 ± 2.65), and medicine use (US $0.11 ± 0.26). The mean patient income loss was US $5.44 ± 6.39 (Table 3).

**Table 3:** Mean Resource Use Cost per Patient per 6 Months to Manage Type 2 Diabetes Mellitus^a^

Cost	Cost Parameter	Overall (481)		Rural (172)		Urban (309)		Mean Differences in US $ (95% CIs)	*P* Value	Comorbid (238)		No Comorbid (243)		Mean Differences in US $ (95% CIs)	*P* Value
		$\bar{x}$	*s*	$\bar{x}$	*s*	$\bar{x}$	*s*			$\bar{x}$	*s*	$\bar{x}$	*s*		
		US$ (NRs)	US$ (NRs)	US$ (NRs)	US$ (NRs)	US$ (NRs)	US$ (NRs)			US$ (NRs)	US$ (NRs)	US$ (NRs)	US$ (NRs)		
Direct medical	Medical consultation	0.51 (60.29)	2.65 (313.15)	0.48 (56.98)	3.43 (406.25)	0.53 (62.61)	2.09 (247.37)	−0.04 (−0.61; 0.52)	0.86	0.42 (49.58)	1.66 (196.11)	0.59 (70.78)	3.35 (395.75)	−0.18 (−0.65; 0.29)	0.46
	Diagnostic	8.55 (1,009.56)	6.40 (756.29)	6.90 (815.41)	6.28 (741.47)	9.46 (1,117.64)	6.29 (743.89)	−2.56 (−3.73; −1.38)	<0.001	8.93 (1,055.04)	6.27 (740.36)	8.17 (965.02)	6.52 (770.49)	0.76 (−0.38; 1.91)	0.192
	Medicine	0.11 (13.39)	0.26 (31.06)	0.11 (12.76)	0.29 (34.42)	0.12 (13.74)	0.25 (29.04)	−0.007 (−0.06; 0.04)	0.77	0.16 (18.77)	0.34 (40.63)	0.06 (7.07)	0.12 (12.85)	0.10 (0.05; 0.14)	<0.001
	Hospitalization	4.84 (571.25)	6.37 (652.98)	3.68 (435.29)	5.32 (628.27)	5.48 (646.93)	6.82 (805.22)	−1.79 (−2.89; −0.69)	0.002	4.61 (544.79)	6.03 (712.19)	5.06 (597.17)	6.70 (791.51)	−0.44 (−1.58; 0.70)	0.45
Direct nonmedical	Transportation	0.61 (71.89)	0.81 (95.63)	0.92 (108.81)	1.02 (120.37)	0.43 (51.33)	0.59 (70.86)	0.49 (0.32; 0.65)	<0.001	0.62 (72.89)	0.82 (97.19)	0.60 (70.92)	0.79 (94.26)	0.02 (−0.13; 0.16)	0.82
	Food items	2.82 (332.86)	2.40 (283.69)	2.92 (353.08)	2.35 (278.04)	2.72 (321.61)	2.43 (286.61)	0.27 (−0.18; 0.72)	0.24	2.97 (350.54)	2.37 (279.74)	2.67 (315.56)	2.43 (287.02)	0.29 (−0.13; 0.73)	0.177
Indirect	Patient income loss	5.44 (643.14)	6.39 (755.30)	4.61 (544.10)	5.44 (642.39)	5.91 (698.27)	6.83 (807.09)	−1.31 (−2.42; −0.19)	0.022	5.23 (617.67)	6.09 (719.42)	5.66 (668.08)	6.68 (789.55)	−0.43 (−1.57; 0.72)	0.47
Total health care cost per patient		22.87 (2,701.86)	16.68 (1,970.44)	19.69 (2,326.43)	15.38 (1,816.69)	24.65 (2,911.66)	17.13 (2,023)	−4.95 (−8.04; −1.86)	0.002	22.93 (2,709.27)	15.33 (1,811.08)	22.81 (2,694.60)	17.94 (2,118.67)	0.12 (−2.87; 3.12)	0.94

*s*, standard deviation; US $, United State dollar (fiscal year 2021).

a *P* value was obtained from independent-sample *t* test.

The total direct expenses of health care resource use of patients living in urban and rural areas was US $24.65 ± 17.13 and US$ 19.69 ± 15.38, respectively. The total direct health care resource use costs per patients with comorbidity and without comorbidity were US $22.93 ± 15.33 and US $22.81 ± 17.94, respectively. The mean income loss of patients living in urban areas (US $5.91 ± 6.83) was higher compared with patients living in rural areas (US $4.61). Similarly, the mean income loss of patients without comorbidity (i.e., US $5.66) was slightly higher compared with patients with comorbidity (US $5.23; Table 3).

**Figure 1:**
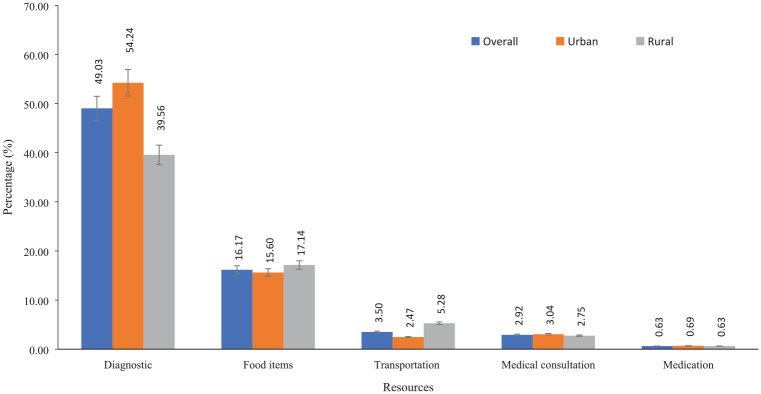
Health care resource use (hospitalization is excluded due to variation in the unit of measurements).

The proportion of diagnosis cost among urban patients accounted for approximately 55% of the total cost, compared with 39.5% in rural patients. However, the cost of food items (i.e., 17.14%) and transportation (5.28%) were higher among rural patients. The cost of medical consultation visits (urban: 3.04%; rural: 2.75%) and medicine use (urban: 0.69%; rural: 0.63%) was higher among urban patients (Figure 1).

Patients who were involved in agriculture or animal husbandry had 27.1% (adjusted β = 0.24, CIs = 0.04; 0.44) higher health care expenses for manage T2D compared with those who were in other occupations (i.e., driver/trained/untrained labor/retired). Patients living in rural areas had 16% (adjusted β = −0.18, CIs = −0.30; −0.05) lower expenses compared with their urban counterparts. Similarly, uninsured patients had 23% (adjusted β = −0.26, CI = −0.38; −0.14) lower expenses to manage and care for T2D compared with their insured counterparts (Table 4).

**Table 4:** Predictive Analysis of Total Cost per Patient^a^

Parameter	Unadjusted Model			Adjusted Model			VIF
	Coefficient (Standard Error)	95% CI	*P* Value	Coefficient (Standard Error)	95% CI	*P* Value	
Intercept	—	—	—	3.34 (0.24)	2.87; 3.81	0.000	
Age	−0.004 (0.003)	−0.01; 0.003	0.26	—	—	—	—
Gender (ref = female)							
Male	0.14 (0.06)	0.02; 0.26	0.021	0.12 (0.09)	-0.05; 0.29	0.175	2.20
Ethnicity (ref = others i.e., Kami/Damai/Sarki/Gaaine/Baadi /Shah/Tharu/Jha)							
Bramin	−0.11 (0.12)	−0.35; 0.14	0.38	−0.22 (0.12)	−0.47; 0.02	0.074	4.45
Newar	−0.21 (0.15)	−0.49; 0.08	0.15	−0.27 (0.14)	−0.56; 0.01	0.059	2.53
Janajati	−0.07 (0.13)	−0.33; 0.19	0.59	−0.17 (0.13)	−0.42; 0.09	0.195	3.69
Chhetri	−0.21 (0.13)	−0.48; 0.05	0.112	−0.22 (0.13)	−0.48; 0.04	0.097	3.26
Marital status (ref = others including unmarried, widowed)							
Married	0.29 (0.12)	0.06; 0.52	0.012	0.13 (0.12)	−0.10; 0.36	0.27	1.08
Education status (ref = Informal)							
No education	−0.29 (0.15)	−0.57; −0.001	0.049	−0.26 (0.14)	−0.55; 0.02	0.065	5.82
Basic (primary and lower secondary)	−0.13 (0.14)	−0.41; 0.16	0.39	−0.22 (0.15)	−0.50; 0.07	0.139	6.27
Intermediate (upper secondary and postsecondary nontertiary)	0.03 (0.18)	−0.31; 0.38	0.85	0.004 (0.18)	−0.34; 0.35	0.98	2.60
Tertiary (university level)	−0.05 (0.18)	−0.41; 0.31	0.79	−0.11 (0.19)	−0.49; 0.27	0.56	2.39
Employment status (ref = others, i.e., driver/ labor (trained/untrained)/retired/household chores)							
Agriculture/animal husbandry	0.10 (0.10)	−0.09; 0.30	0.30	0.24 (0.10)	0.04; 0.44	0.020	3.04
Housewife	0.01 (0.11)	−0.21; 0.22	0.97	0.23 (0.13)	−0.02; 0.48	0.067	3.38
Business	0.11 (0.12)	−0.12; 0.34	0.36	0.14 (0.12)	−0.09; 0.37	0.24	2.12
Services	0.19 (0.14)	−0.08; 0.46	0.163	0.15 (0.14)	−0.12; 0.43	0.28	1.73
Religion (ref = Christian)							
Hindu	0.02 (0.18)	−0.33; 0.37	0.92	—	—	—	—
Buddhist	−0.25 (0.21)	−0.66; 0.15	0.22	—	—	—	—
Residency (ref = urban living)	−0.22 (0.06)	−0.35; −0.10	<0.001	−0.18 (0.06)	−0.30; −0.05	0.005	1.14
Comorbidity (ref = no comorbid patients)	−0.01 (0.06)	−0.12; 0.11	0.93	—	—	—	—
Year of living with T2DM (ref = >1 y)	−0.09 (0.11)	−0.30; 0.13	0.42	—	—	—	—
Insurance (ref = yes)	−0.32 (0.06)	−0.44; −0.20	<0.001	−0.26 (0.06)	−0.38; −0.14	<0.001	1.11
Monthly family income quintile (US $), *n* (%) (ref = richest quintile)							
Poorest (≤84.65)	−0.26 (0.10)	−0.45; −0.07	0.007	−0.15 (0.10)	−0.34; 0.04	0.131	1.65
Second quintile (84.66–126.98)	−0.08 (0.09)	−0.25; 0.09	0.36	−0.001 (0.09)	−0.18; 0.18	0.99	1.75
Third quintile (126.99–228.56)	0.02 (0.09)	−0.17; 0.20	0.85	0.09 (0.09)	−0.10; 0.27	0.37	1.56
Fourth quintile (228.57–423.26)	0.04 (0.09)	−0.15; 0.23	0.68	0.04 (0.09)	−0.14; 0.23	0.64	1.54

—, not applicable; T2DM, type 2 diabetes mellitus; VIF, variance inflation factor.

a *P* value was obtained from the generalized linear model; covariants with *P* values of ≤0.20 in the unadjusted model were added in the adjusted model; multicollinearity of independent variables was identified. The accepted cutoff for VIF was <5.

The results of the sensitivity analysis are presented in Table 5 and show that patients living in rural areas had 15% (adjusted beta β = −0.16, CIs = −0.30; −0.02) lower health care expenses compared with their urban counterparts. Uninsured patients had 24% (adjusted beta β = −0.27, CI = −0.39; −0.14) lower health care expenses compared with their insured counterparts (Table 5).

**Table 5:** Sensitivity Analysis Testing the Robustness of the Results for Total Health Care Resource Use Cost

Parameter	Unadjusted Model^a^			Adjusted Model^a^		
	Coefficient (Standard Error)	95% CI	*P* Value	Coefficient (Standard Error)	95% CI	*P* Value
Intercept	—	—	—	3.51 (0.20)	3.11; 3.91	<0.001
Age	−0.004 (0.003)	−0.01; 0.003	0.26	—	—	—
Gender (ref = female)						
Male	0.14 (0.06)	0.01; 0.27	0.032	0.07 (0.07)	−0.08; 0.21	0.37
Ethnicity (ref = others, i.e., Kami/Damai/Sarki/Gaaine/Baadi/Shah/Tharu/Jha)						
Bramin	−0.11 (0.13)	−0.35; 0.16	0.40	−0.19 (0.12)	−0.42; 0.05	0.121
Newar	−0.21 (0.14)	−0.48; 0.08	0.14	−0.26 (0.14)	−0.54; 0.03	0.067
Janajati	−0.07 (0.13)	−0.32; 0.20	0.59	−0.16 (0.12)	−0.40; 0.08	0.185
Chhetri	−0.21 (0.15)	−0.50; 0.09	0.158	−0.20 (0.13)	−0.45; 0.08	0.142
Marital status (ref = others including unmarried, widowed)						
Married	0.29 (0.09)	0.12; 0.47	<0.001	0.13 (0.10)	−0.07; 0.32	0.187
Education status (ref = Informal)						
No education	−0.29 (0.11)	−0.48; -0.07	0.008	−0.25 (0.13)	−0.49; 0.001	0.044
Basic (primary and lower secondary)	−0.13 (0.10)	−0.31; 0.09	0.21	−0.20 (0.12)	−0.42; 0.05	0.093
Intermediate (upper secondary and postsecondary nontertiary)	0.03 (0.15)	−0.24; 0.32	0.82	−0.01 (0.16)	−0.31; 0.32	0.97
Tertiary (university level)	−0.05 (0.20)	−0.43; 0.33	0.80	−0.15 (0.21)	−0.56; 0.25	0.46
Employment status (ref = others, i.e., driver/labor (trained/untrained)/retired/household chores)						
Agriculture/animal husbandry	0.10 (0.09)	−0.07; 0.29	0.25	—	—	—
Housewife	0.01 (0.10)	−0.19; 0.21	0.96	—	—	—
Business	0.11 (0.11)	−0.11; 0.32	0.32	—	—	—
Services	0.19 (0.17)	−0.15; 0.52	0.27	—	—	—
Religion (ref = Christian)						
Hindu	0.02 (0.20)	−0.32; 0.46	0.93	—	—	—
Buddhist	−0.25 (0.22)	−0.64; 0.22	0.22	—	—	—
Residency (ref = urban living)	−0.22 (0.07)	−0.36; −0.09	0.001	−0.16 (0.07)	−0.30; −0.02	0.027
Comorbidity (ref = no comorbid patients)	-0.01 (0.07)	−0.14; 0.13	0.94	—	—	—
Year of living with T2DM (ref = above 1 year)	−0.09 (0.13)	−0.33; 0.16	0.48	—	—	—
Insurance (ref = yes)	−0.32 (0.07)	−0.45; −0.19	<0.001	−0.27 (0.06)	−0.39; −0.14	<0.001
Monthly family income quintile (US $), *n* (%) (ref = richest quintile)						
Poorest (≤84.65)	−0.26 (0.11)	−0.47; -0.06	0.016	−0.13 (0.10)	−0.32; 0.06	0.181
Second quintile (84.66–126.98)	−0.08 (0.09)	−0.26; 0.10	0.37	0.01 (0.09)	−0.15; 0.18	0.90
Third quintile (126.99–228.56)	0.02 (0.12)	−0.22; 0.25	0.88	0.11 (0.12)	−0.12; 0.33	0.38
Fourth quintile (228.57–423.26)	0.04 (0.09)	−0.14; 0.22	0.67	0.05 (0.09)	−0.11; 0.23	0.55

—, not applicable; CI, confidence interval; T2DM, type 2 diabetes mellitus.

a Ten thousand bootstraps resampling. The *P* value was obtained from the generalized linear model; covariants with *P*≤0.20 in the unadjusted model were added in the adjusted model.

## Discussion

This study provides comprehensive evidence in relation to health care expenses for managing T2D in community settings in Nepal. The current study indicated that T2D imposed a substantial economic burden on patients and the health care system in Nepal in terms of resource use and their associated costs. Moreover, health care expenses were particularly high among patients living in urban areas and patients with comorbidities. This study further demonstrates that certain demographic and socioeconomic characteristics such as occupation (i.e., agriculture/animal husbandry), residency status, and insurance status of patients had a significant association with health care expenses to manage T2D.

The current estimated per-patient cost of T2D was lower that previously estimated by a systematic review focusing on low- and middle-income countries where the cost per patient ranged from US $5 to $40 per visit.^26^ This review also reported a cost per visit of US $4.39, laboratory cost of US $31.70 per year, and medicine cost of US $77.70 per year in Nepal in 2019. Among the limited cost of T2D studies in Nepal, Kattel et al.^27^ estimated that the average cost for medication and screening of diabetes was US $103 per year, and the mean indirect cost was US $102 per year. However, these estimates are different from those reported in the current study, because the study by Kattel et al. reported the cost based on annual resource use in a hospital setting. In addition, the authors also included both type 1 and 2 diabetes in a hospital. In contrast, the current study was focused solely on health care resource use within 6 mo among the patients with T2D only in a community setting.

T2D imposed a significantly higher financial burden among patients who resided in the urban area of the selected districts. The total cost for diabetes management among the urban patients was about 20% higher than the cost for patients living in the rural areas, and it was almost 8.5% of the average monthly family income of patients. Furthermore, in the current study, the reported loss of income of urban patients seemed to be higher than that of rural patients. However, the transportation costs for the rural patients were higher than for urban patients. Our estimate is lower than a previously conducted hospital-based study by Shrestha and Shakya^8^ in Kathmandu (i.e., the capital city of Nepal), who found that the total cost to manage diabetes was US $147.61per patient. This is because the costs reported by Shrestha and Shakya were based on the selected hospitals of the main city in Nepal, which has more facilities and expenses. Moreover, the expenditure was calculated based on the patient’s self-reported data. Conversely, the current study was based on the community setting; the urban settings within the selected districts were different in terms of resources compared with the Kathmandu district, and the expenditure was calculated based on secondary sources such as charges of Dhulikhel Hospital, transportation costs from Nepal’s government’s pricing list, and food items from the available resources. The higher cost in urban patients might be due to a higher volume of resource use, changes in the mode of transportation, changes in diet due to access to fast food restaurants and multinational supermarkets, and changes to a more sedentary lifestyle compared with the rural setting.^28^ In contrast, in rural settings, the limited availability of resources such as specialized services, diagnosis, inpatient hospital stays, advanced technologies, lower administrative costs, health services focused on prevention rather than curing, and stronger community and social support networks (i.e., people taking care of one another informally) may explain the lower expenditure to manage T2D. Furthermore, the lower cost of living in rural areas compared with urban areas, especially in relation to housing, might have played a significant role in causing lower health care expenditure for rural patients.^29–31^ In contrast, a previously published systematic review in US-based populations reported that the cost and burden of T2D is higher among the rural population.^32^ However, as a high-income country, the United States is very different from Nepal in terms of facilities/services, resources use, mode of transportation, and level of T2D knowledge, which makes comparisons difficult.

The present study also estimated the volume of resources used and their associated costs in terms of the presence of comorbidities. Comorbid patients with T2D accounted for higher health care resource use cost compared with patients without comorbidity. Previous studies^33,34^ in India reported that the cost of T2D care with complications was higher compared with care without complications, which is in line with the current study. Moreover, a cross-sectional study in China also reported that the cost implication was less among diabetes patients with fewer complications.^35^ As such, more comorbidities pose a greater economic burden for patients.^36,37^ This might be due to the increasing number of hospital visits, duration of hospital stays, medications, and health care services with comorbidities.^35,38^ However, a previous study conducted in the United States reported that the expenses of Medicare patients with T2D were not different based on comorbid conditions.^39^ This difference may be attributed to the age of included participants in the US study (i.e., 65 y and older), and the type of comorbidity was identified based on codes from health insurance claims data (using the International Classification of Diseases [ICD-9]). However, the participants of the current study were aged 30 to 70 y with clinically diagnosed with T2D, and their comorbidities were self-reported.

The study has some limitations in the cost estimation to manage T2D. First, the current study is based on the resource use within 6 mo of duration among a small sample in the selected district of Nepal. This might not be sufficient to generalize the findings to the whole nation. Second, the cost calculation was done based on secondary sources such as health specialist visits, medication, and hospitalization charges of Dhulikhel Hospital, transportation cost from Nepal’s government’s pricing list, and food items from the available resources, which may under- or overestimate the actual cost to a specific patient. Third, the pattern of resource use and its prices may change over time, which may alter the total cost estimate. Finally, the cost from a societal perspective is lacking in the current study due to the unavailability of data on absenteeism, presentism, and the cost of the patient’s caregivers.

Despite these limitations, this first costing study estimated that the cost to manage T2D is significantly higher in the context of Nepal, in which medications, medical consultation, screening, and hospitalization fees are less likely to be funded by the government sector and coverage of health insurance is not ideal. Furthermore, there is a lack of health care facilities, treatment is unaffordable, and there are limited national guidelines for health care workers for the management of T2D in Nepal. However, international guidelines are well established but are missing in the implementation of health care facilities of Nepal.^40^ Therefore, the present study provides useful insights for policy makers and health care providers with regard to the future resource use and costs to manage T2D. However, more evidence is needed to obtain a broader picture of the direct and indirect cost of health care resource use among diabetes patients in the wider community.

To conclude, T2D imposes a substantial financial burden to the health care system and individuals. These high expenses highlight the importance of establishing innovative strategies for financial protection for T2D patients. Moreover, the increasing expenses among urban patients underscores the significance of a comprehensive diabetes care plan and efforts focusing on health behavior change programs in the community. In addition, effective health behavior interventions that manage, care, and delay the development of T2D and its comorbidity might result in substantial cost savings. Thus, the current cost estimation can be used as one of the essential measures for developing T2D prevention and control strategies and it will be beneficial for the policy makers, academics, and civil society of developing nations such as Nepal.

## Supplemental Material

sj-docx-1-mpp-10.1177_23814683231216938 – Supplemental material for Estimating the Health Care Expenditure to Manage and Care for Type 2 Diabetes in Nepal: A Patient Perspective Click here for additional data file. Supplemental material, sj-docx-1-mpp-10.1177_23814683231216938 for Estimating the Health Care Expenditure to Manage and Care for Type 2 Diabetes in Nepal: A Patient Perspective by Padam Kanta Dahal, Lal Rawal, Zanfina Ademi, Rashidul Alam Mahumud, Grish Paudel and Corneel Vandelanotte in MDM Policy & Practice

## References

[1] Khan MAB Hashim MJ King JK Govender RD Mustafa H Al Kaabi J . Epidemiology of type 2 diabetes—global burden of disease and forecasted trends. J Epidemiol Glob Health. 2020;10(1):107–11. DOI: 10.2991/jegh.k.191028.001 PMC7310804 32175717

[2] International Diabetes Federation. IDF Diabetes Atlas 10th Edition. Brussels (Belgium): International Diabetes Federation; 2021.

[3] Bommer C Heesemann E Sagalova V , et al. The global economic burden of diabetes in adults aged 20-79 years: a cost-of-illness study. Lancet Diabetes Endocrinol. 2017;5(6):423–30. DOI: 10.1016/S2213-8587(17)30097-9 10.1016/S2213-8587(17)30097-9 28456416

[4] Mattei J Malik V Wedick NM , et al. Reducing the global burden of type 2 diabetes by improving the quality of staple foods: the global nutrition and epidemiologic transition initiative. Global Health. 2015;11(1):23. DOI: 10.1186/s12992-015-0109-9 26040275 PMC4489001

[5] Shrestha N Karki K Poudyal A , et al. Prevalence of diabetes mellitus and associated risk factors in Nepal: findings from a nationwide population-based survey. BMJ Open. 2022;12(2):e060750. DOI: 10.1136/bmjopen-2022-060750 PMC8867329 35193925

[6] International Diabetes Federation. IDF Diabetes Atlas 10th Edition 2021; Nepal Diabetes Report 2000-2045. Brussels (Belgium): International Diabetes Federation; 2021.

[7] World Health Organisation. Global health expenditure database. 2021. Available from: https://apps.who.int/nha/database/country_profile/Index/en [Accessed 10 October, 2021].

[8] Shrestha R Shakya A. Health Expenditure Among the Outpatient of Type-2 Diabetes in Selected Hospital of Kathmandu District: A Cross Sectional Study. Laurel Hollow (NY): Cold Spring Harbor Laboratory; 2021.

[9] Naing L Nordin RB Abdul Rahman H Naing YT. Sample size calculation for prevalence studies using Scalex and ScalaR calculators. BMC Med Res Methodol. 2022;22(1):209. DOI: 10.1186/s12874-022-01694-7 35907796 PMC9338613

[10] Husereau D Drummond M Augustovski F , et al. Consolidated Health Economic Evaluation Reporting Standards (CHEERS) 2022 explanation and elaboration: a report of the ISPOR CHEERS II Good Practices Task Force. Value Health. 2022;25(1):10–31. DOI: 10.1016/j.jval.2021.10.008 35031088

[11] Laberge M Coulibaly LP Berthelot S , et al. Development and validation of an instrument to measure health-related out-of-pocket costs: the cost for patients questionnaire. Value Health. 2021;24(8):1172–81. DOI: 10.1016/j.jval.2021.03.016 34372983

[12] Curtis LA Burns A. Unit Costs of Health and Social Care 2019. Canterbury (UK): Personal Social Services Research Unit, University of Kent; 2019.

[13] Cuschieri S. The STROBE guidelines. Saudi J Anaesth. 2019;13(suppl 1):S31–4. DOI: 10.4103/sja.SJA_543_18 PMC6398292 30930717

[14] The World Bank. DataBank, World development indicators. 2022. Available from: https://databank.worldbank.org/reports.aspx?source=2&series=PA.NUS.FCRF&country=# [Accessed 13 Febuary, 2022].

[15] Wang PY Fang JC Gao ZH Zhang C Xie SY. Higher intake of fruits, vegetables or their fiber reduces the risk of type 2 diabetes: a meta-analysis. J Diabetes Investig. 2016;7(1):56–69. DOI: 10.1111/jdi.12376 PMC4718092 26816602

[16] NUMBEO. Food prices in Nepal. 2022. Available from: https://www.numbeo.com/food-prices/country_result.jsp?country=Nepal [Accessed 25 Febuary, 2022].

[17] Slavin JL Lloyd B. Health benefits of fruits and vegetables. Adv Nutr. 2012;3(4):506–16. DOI: 10.3945/an.112.002154 PMC3649719 22797986

[18] American Heart Association. Fruits and vegetables serving sizes infographic. 2022. Available from: https://www.heart.org/en/healthy-living/healthy-eating/add-color/fruits-and-vegetables-serving-sizes [Accessed 27 Febuary, 2022].

[19] Nutrition Australia. Australian dietary guidelines: standard serves. 2014. Available from: https://nutritionaustralia.org/fact-sheets/adgs-standard-serves/#Standard-serves-of-each-food-group [Accessed 12 March, 2022].

[20] Ministry of Physical Infrastructure and Transport Government of Nepal, Department of Transport Management. Vehicle fare. 2022. Available from: https://www.dotm.gov.np/download-content/vehicle-fare-rates [Accessed 21 Febuary, 2022].

[21] Human capital approach. In: Kirch W , ed. Encyclopedia of Public Health. Dordrecht: Springer Netherlands; 2008. p 697–8.

[22] Ministry of Labour Government of Nepal, Employement and Social Security. Minimum monthly wages/salary in Nepal 2078/2021 Set. 2021. Available from: https://drive.google.com/file/d/1bPunPvT6dCml-jOIYtOn5sEaregDeN7h/view [Accessed January, 2022].

[23] Chapko MK Liu CF Perkins M Li YF Fortney JC Maciejewski ML. Equivalence of two healthcare costing methods: bottom-up and top-down. Health Econ. 2009;18(10):1188–201. DOI: 10.1002/hec.1422 19097041

[24] Potter S Davies C Davies G Rice C Hollingworth W. The use of micro-costing in economic analyses of surgical interventions: a systematic review. Health Econ Rev. 2020;10(1):3. DOI: 10.1186/s13561-020-0260-8 31997021 PMC6990532

[25] Kulesa A Krzywinski M Blainey P Altman N. Sampling distributions and the bootstrap. Nat Methods. 2015;12(6):477–8. DOI: 10.1038/nmeth.3414 PMC4737599 26221652

[26] Moucheraud C Lenz C Latkovic M Wirtz VJ. The costs of diabetes treatment in low- and middle-income countries: a systematic review. BMJ Glob Health. 2019;4(1):e001258. DOI: 10.1136/bmjgh-2018-001258 PMC6407562 30899566

[27] Kattel V Subedi M Agrawal Y , et al. Cost of illness study of regular out patient department diabetic in a low income country: a cross sectional study. J Diabetes Endocrinol Assoc Nepal. 2019;3(1):16–25. DOI: 10.3126/jdean.v3i1.24060

[28] Gassasse Z Smith D Finer S Gallo V. Association between urbanisation and type 2 diabetes: an ecological study. BMJ Glob Health. 2017;2(4):e000473. DOI: 10.1136/bmjgh-2017-000473 PMC5663267 29104770

[29] Zhang T Liu C Ni Z. Association of access to healthcare with self-assessed health and quality of life among old adults with chronic disease in China: urban versus rural populations. Int J Environ Res Public Health. 2019;16(14):2592. DOI: 10.3390/ijerph16142592 31330818 PMC6679116

[30] Zhang X Dupre ME Qiu L Zhou W Zhao Y Gu D. Urban-rural differences in the association between access to healthcare and health outcomes among older adults in China. BMC Geriatr. 2017;17(1):151. DOI: 10.1186/s12877-017-0538-9 28724355 PMC5516359

[31] Chuma J Gilson L Molyneux C. Treatment-seeking behaviour, cost burdens and coping strategies among rural and urban households in Coastal Kenya: an equity analysis. Trop Med Int Health. 2007;12(5):673–86. DOI: 10.1111/j.1365-3156.2007.01825.x 17445135

[32] Dugani SB Mielke MM Vella A. Burden and management of type 2 diabetes in rural United States. Diabetes Metab Res Rev. 2021;37(5):e3410. DOI: 10.1002/dmrr.3410 PMC7990742 33021052

[33] Tharkar S Devarajan A Kumpatla S Viswanathan V. The socioeconomics of diabetes from a developing country: a population based cost of illness study. Diabetes Res Clin Pract. 2010;89(3):334–40. DOI: 10.1016/j.diabres.2010.05.009 20538363

[34] Akari S Mateti UV Kunduru BR. Health-care cost of diabetes in South India: a cost of illness study. J Res Pharm Pract. 2013;2(3):114–7. DOI: 10.4103/2279-042x.122382 PMC4076919 24991617

[35] Wang W Fu C Zhuo H Luo J Xu B. Factors affecting costs and utilization of type 2 diabetes healthcare: a cross-sectional survey among 15 hospitals in urban China. BMC Health Serv Res. 2010;10(1):244. DOI: 10.1186/1472-6963-10-244 20727137 PMC2936377

[36] Afroz A Alramadan MJ Hossain MN , et al. Cost-of-illness of type 2 diabetes mellitus in low and lower-middle income countries: a systematic review. BMC Health Serv Res. 2018;18(1):972. DOI: 10.1186/s12913-018-3772-8 30558591 PMC6296053

[37] Lin P-J Pope E Zhou FL. Comorbidity type and health care costs in type 2 diabetes: a retrospective claims database analysis. Diabetes Ther. 2018;9(5):1907–18. DOI: 10.1007/s13300-018-0477-2 PMC6167298 30097994

[38] Jacobs J Sena M Fox N. The cost of hospitalization for the late complications of diabetes in the United States. Diabet Med. 1991;8 Spec No:S23–9. DOI: 10.1111/j.1464-5491.1991.tb02151.x 1825950

[39] Halanych JH Safford MM Keys WC , et al. Burden of comorbid medical conditions and quality of diabetes care. Diabetes Care. 2007;30(12):2999–3004. DOI: 10.2337/dc06-1836 17717287

[40] Shrestha D Shrestha P Sharma S , et al. National consensus statement on the management of type 2 diabetes mellitus in Nepal. J Diabetes Endocrinol Assoc Nepal. 2019;3:38–57. DOI: 10.3126/jdean.v3i1.24072

